# Potential anti-proliferative activity of *Salix mucronata* and *Triticum spelta* plant extracts on liver and colorectal cancer cell lines

**DOI:** 10.1038/s41598-023-30845-z

**Published:** 2023-03-07

**Authors:** Ghada M. Ahmad, Marwa M. Abu Serie, Mohamed S. Abdel-Latif, Tayseer Ghoneem, Doaa A. Ghareeb, Galila A. Yacout

**Affiliations:** 1 Department of Medical Laboratory Technology, Faculty of Applied Health Sciences Technology, Pharos University in Alexandria, Alexandria, Egypt; 2 Medical Biotechnology Department, Genetic Engineering and Biotechnology Research Institute (GE-BRI), City of Scientific Research and Technological Applications (SRTA-City), New Borg El-Arab, 21934 Alexandria Egypt; 3 Department of Biochemistry, Faculty of Science, Alexandria University, Alexandria, Egypt

**Keywords:** Biochemistry, Cancer, Drug discovery, Molecular biology

## Abstract

Cancer’s etiology is linked to oxidative stress. As a result, it's vital to find effective natural antioxidant remedies. *Salix mucronata* and *Triticum spelta* plant extracts were prepared using five different solvents and examined for their cytotoxicity against liver HepG2 cancer cell line. It was found that *Salix mucronata* ethanolic extract is high in antioxidant mediated anti-cancer activity. The functional constituents (phenolic and flavonoids) as well as preparation of different ethanolic concentrations used to study their properties that include DPPH, oxygen, hydroxyl, nitrogen radical scavenging activities, ferric reducing power and metal chelating activities. The MTT assay was used to determine antioxidant-mediated anti-cancer activity against human liver (HepG2) and colorectal (Caco-2) cancer cells to calculate the half-maximal growth inhibitory concentration (IC_50_). Moreover, flow cytometry analysis was used to quantify the apoptotic effect on the treated cancer cells. Additionally, qRTPCR of *p53*, *BCL2*, *Cyclin D*, *MMP9* and *VEGF* were measured. Furthermore, HPLC was used to assess the most effective ingredients of the plant extract. *Salix mucronata* 50% ethanol extract had the highest polyphenolic content, anti-oxidant, and anti-proliferative activity. *Salix mucronata* increased the number of total apoptotic cells, and caused an upregulation of *p53* gene expression by more than five folds and a downregulation of gene expression level of *BCL2*, *Cyclin D*, *MMP9* and *VEGF* by more than five folds. Consequently, that could modulate oxidative stress and improve the effectiveness of cancer therapy. Results, also, showed that *Triticum spelta* ethanolic extract was less effective than *Salix mucronata*. Therefore, *Salix mucronata* ethanolic extract represents promising surrogate natural therapy for apoptosis-mediated cancer and recommended for further investigation using animal model.

## Introduction

In terms of cancer mortality worldwide, hepatocellular carcinoma is the second most frequent malignancy. Hepatocellular carcinoma is caused by chronic liver illness, which is brought on by a number of risk factors; including, chronic infections with the hepatitis B and C viruses (HBV and HCV) and risk factors like co-infection with the hepatitis D virus (HDV), alcohol use, cigarette smoking, aflatoxin exposure in the environment, tainted water from blue-green algal toxin, and betel nut consumption.

Chronic liver damage from ethanol consumption and metabolic syndrome eventually results in steatosis, steatohepatitis, cirrhosis, and hepatocellular carcinoma^1^. The majority of the initial genetic events that cause hepatocellular carcinoma are still unknown, despite the fact that gene sequencing studies have identified numerous genes associated with the disease^2^. Hepatocellular carcinoma has been linked to genomic instability, such as chromosomal or single nucleotide polymorphism, recurrent somatic mutations in genes such as the *TERT* promoter, *p53*, *CTNNB1*, *ARID1A*, and *FGF* as well as signaling pathways such as *JAK/STAT*, *WntB-catenin*, and *PI3K-AKT-mTOR*. The genetic heterogeneity of hepatocellular carcinoma is most likely the reason why no possible targeted therapy has been identified. The *p53* gene mutation and *Ki-67* protein expression are the traditional prognostic indicators for hepatocellular carcinoma, and both have frequently been shown to be associated with a poor prognosis^3^.

The second most lethal cancer is the colorectal cancer (CRC), which is also the most frequent diagnosed disease. Adenomatous polyps, polyps with villous or tubule-villous dysplasia, genetic risk factors, and environmental linkages, all, are indicating to a high probability of synchronous and metachronous CRC primary malignancy. It is well recognized that CRC and inflammatory bowel disease (IBD), namely ulcerative colitis, are related to each other^4^. Chromosomal instability, mismatch repair, and hypermethylation are the three main molecular processes associated with CRC^5^. If the problem present in the ileocolic area, Crohn's disease may make CRC riskier. It takes a buildup of genetic abnormalities, either somatic (acquired) or germline, for the normal colonic epithelium to develop into a precancerous lesion (adenoma) and finally into invasive carcinoma (inherited)^5^. The hypothesis of colonic carcinogenesis describes the evolution of clonal mutations that give cells the ability to live forever and allow for the development of further mutations that result in additional cancer hallmarks like proliferation, invasion, and metastasis^6^.

Furthermore, the administration of existing chemotherapeutic drug is limited by the complicated pathophysiological pathways of cancer, substantial side effects, and poor prognoses. In order to simultaneously target the main dysregulated signaling mediators in the genesis of cancer while having fewer side effects, innovative safe natural therapeutic medicines must be developed.

The plant kingdom is described in this phrase as a rich source of useful compounds. Plant-produced secondary metabolites have the potential to control a number of dysregulated pathways in cancer.

Natural phytochemical anti-cancer polyphenolic compounds derived from plant; attracted interest as a potential replacement therapy^7^. Phenolic' anti-carcinogenic effects are linked to their capacity to reduce cell proliferation by inhibiting extracellular signal-regulated kinase (*ERK1/2*), *Cyclin D*, cyclin-dependent kinases (*CDKs*), angiogenic factors (like *VEGF* and *MIC-1*), oncogenic signaling cascades (like *PI3K* and *Akt*), apoptosis, and inhibition of cellular migration and metastasis^8^.

Flavonoids are optimistic phytochemicals among the secondary metabolites since they have well-established biological functions and few negative consequences. Flavonoids decrease a substantial dysregulated pathway in cancer by inhibiting B-cell lymphoma 2 (*Bcl-2*) via the *P53* signaling pathway, which is a significant apoptotic target in many cancer types^9^. Two edible plants, *Salix mucronata* and *Tritticum spelta*; were tested for their apoptotic-mediated anticancer activity of antioxidant-rich extracts using different fraction to find the suitable one that would be more effective against liver and colorectal cancer cell lines.

*Salix mucronata* and *Tritticum spelta* contain phenolic compounds, flavonoids, terpenes and lignans. The phenolic compounds isolated, were the most abundant with reported analgesic, antipyretic, anti-inflammatory and anti-rheumatic properties. Salicin was the main anti-leukemia component found in *Salix mucronata* leaves and could be dissolved in both water and ethanol. Salicin may have an anti-tumor impact. However, other metabolites may boost the efficacy of the *Salix mucronata* extract when compared to pure salicin^10^. *Tritticum spelta* has a macronutrient and mineral content such as fiber, lipid, protein, phenolic compounds, iron, zinc, copper, magnesium, potassium, sodium, and selenium^11^.

Spelt has a high nutritional value and can decrease cholesterol. However, there is no scientific evidence to support such evidence. In light of their physiological effects, phytosterols, which have not been investigated in this crop, may contribute to the qualities of spelt^12^. According to descriptions of phytosterols as bioactive chemicals, they may offer defense against some cancer forms and have immuno-modulating properties^13^. Cereals contain free sterols (FS) and four types of conjugated sterols: steryl fatty acid esters (SE), hydroxycinnamate steryl esters (HSE), steryl glycosides (SG), and acylated steryl glycosides (ASG)^12,13^. The majority of the sterols in cereals are unsaturated, but there are also some saturated sterols, known as stanols^12^.

In the present study, authors investigated in vitro anti-proliferative effect of polyphenolic and flavonoids content that exist in two edible natural plants (*Salix mucronata* and *Tritticum spelta*) that can influence tumor protein *p53*, *BCL2*, *Cyclin D*, *MMP9* and *VEGF* signaling pathway and highlight various mechanisms by which this can happen against liver and colon cancer cell lines.

## Results

### Phytochemicals and total antioxidant contents

**Figure 1:**
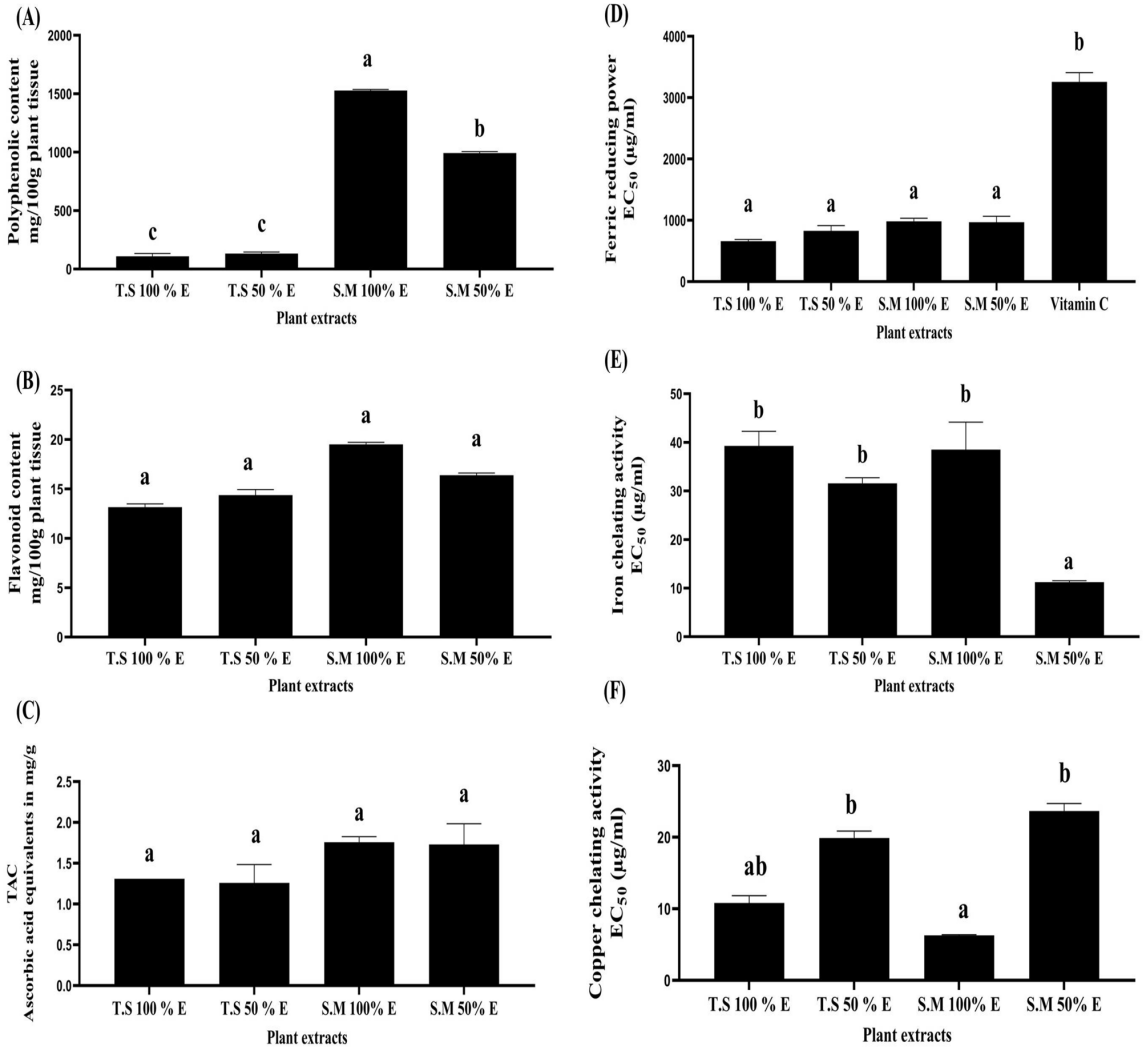
Phyto-chemicals and total antioxidant contents as well as iron reducing and metal chelating potentials in each examined ethanolic plant extract. (**A**) Phenolic content (mg/100 g plant tissue), (**B**) Flavonoid content (mg/100 g plant tissue), and (**C**) Total antioxidant content (ascorbic acid equivalents in mg/g). (**D**) Ferric reducing power (EC_50_: µg/mL) (**E**) Iron chelating activity (EC_50_: µg/mL) and (**F**) Copper chelating activity (EC_50_: µg/mL). All data are expressed as mean ± SEM. Different letters are significantly different at p ≤ 0.05.

Results in Fig. 1A showed that *Salix mucronata* 100% ethanolic extract (S.M 100% EE) significantly had the highest phenolic content followed by *Salix mucronata* 50% ethanolic extract (S.M 50% EE) (1527.34 ± 10.2 and 992.6 ± 12.2 mg/100 g plant tissue, respectively) among all examined plant extracts. It was found that *Tritticum spelta* 100% and 50% ethanolic extract (T.S 100% and 50% EE), non-significantly, had the lowest phenolic content (108.97 ± 24.48 and 132.44 ± 13.26 mg/100 g plant tissue, respectively).

As observed in Fig. 1B; all examined ethanolic plant extracts contain nearly a considerable concentration of the flavonoid content. Flavonoid content arranged as follow; S.M 100% EE are more than in S.M 50% EE followed by T.S 50% EE and finally T.S 100% EE (19.5 ± 0.2, 16.38 ± 0.2, 14.38 ± 0.5 and 13.15 ± 0.3 mg/100 g plant tissue, respectively). Therefore, the presence of high phytochemical content resulted in non-significant higher total anti-oxidant capacity in S.M 100% EE followed by S.M 50% EE, T.S 100% EE and T.S 50% EE (1.75 ± 0.07, 1.73 ± 0.25, 1.310 ± 0 and 1.26 ± 0.225, ascorbic acid equivalents in mg/g, respectively) as shown in Fig. 1C.

According to ferric reducing power showed in Fig. 1D; T.S 100% EE showed non-significant higher ferric reducing power than T.S 50% EE, S.M 50% EE and S.M 100% EE; that significantly reflects the lowest EC_50_ values for each corresponding plant extract (658.75 ± 28.09, 827.04 ± 88.7, 967.6 ± 98.3 and 981.1 ± 54 µg/mL, respectively). All examined plant extracts were more potent than vitamin C (3255.52 ± 151.7 µg/mL).

In Fig. 1E; the highest iron chelating activity were significantly recorded in S.M 50% EE (11.21 ± 0.31 µg/mL) while the other examined plant extract, non-significant, showed the same activity according to their EC_50_ (µg/mL) and they were arranged according to their EC_50_ as follow; T.S 100% EE, S.M 100% EE and T.S 50% EE (39.2 ± 3, 38.5 ± 5.6 and 31.5 ± 1.18 µg/mL, respectively).

Figure 1F demonstrated, non-significantly, the highest copper chelating activity with the lowest EC_50_ that recorded in S.M 100% EE followed by T.S 100% EE; on the contrary, it was significant with T.S 50% EE and S.M 50% EE (6.2 ± 0.09, 10.8 ± 1, 19.9 ± 0.95 and 23.66 ± 1.03 µg/mL, respectively).

### Anti-oxidant activities of each plant extract and heat map analysis

**Figure 2:**
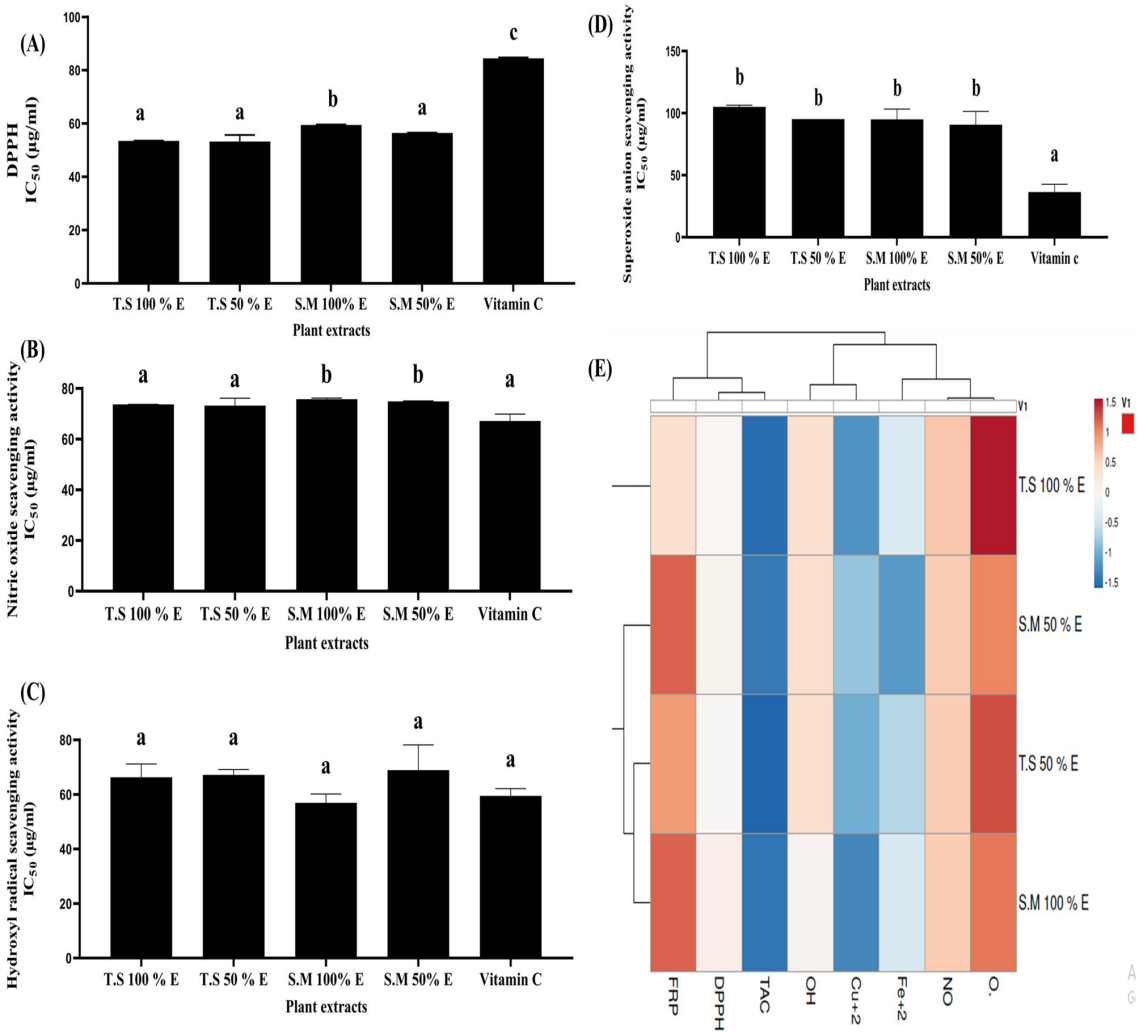
The radical scavenging activity of each plant extract in the term of IC_50_ (µg/mL) with illustrative heat map distribution. (**A**): DPPH scavenging activity (IC_50_: µg/mL), (**B**) NO scavenging activity (IC_50_: µg/mL), (**C**) OH scavenging activity (IC_50_: µg/mL), and (**D**) Superoxide anion scavenging activity (IC_50_: µg/mL). All data are expressed as mean ± SEM. Different letters are significantly different at p ≤ 0.05. (**E**) Heat map distribution of in vitro antioxidant activities of the studied extracts. The color distributed from white (low level antioxidant activity or content) to blue (high level anti-oxidant activity or content).

Figure 2 illustrates the antioxidant capabilities of the examined extracts. In Fig. 2A, The present results of DPPH scavenging activities of all examined plants extracts revealed that IC_50_ value of T.S 50% EE is, non-significantly, lower than T.S 100% EE, followed by S.M 50% EE (53.22 ± 2.48, 53.49 ± 0.079 and 56.46 ± 0.113, µg/mL, respectively). IC_50_ of T.S 50% and 100% EE, and S.M 50% EE were, significantly, slightly decreased than IC_50_ of S.M 100% EE and vitamin C (59.44 ± 0.19 and 84.44 ± 0.35 µg/mL, respectively). In Fig. 2B; The nitric oxide scavenging activities of each examined extract showed non-significant lowest IC_50_ values in vitamin C followed by T.S 50% EE and T.S 100% EE (67.14 ± 2.77, 73.2 ± 2.95 and 73.7 ± 0.083 µg/mL, respectively); and, on the opposite, it is significant with S.M 50% EE and S.M 100% EE (75.7 ± 0.4 and 74.8 ± 0.19 µg/mL, respectively).

Figure 2C showed that all plant extracts demonstrated non-significant highest hydroxyl radical scavenging abilities. IC_50_ values of the examined extracts arranged from the lowest to the highest value as follow; S.M 100% EE, vitamin C, T.S 100% EE, T.S 50% EE and S.M 50% EE (56.8 ± 3.2, 59.4 ± 2.6, 66.3 ± 4.9, 67.16 ± 1.9 and 68.9 ± 9.24 µg/mL, respectively).

As shown in Fig. 2D, the strong superoxide anion scavenging abilities were significantly noticed in vitamin C (36.44 ± 6.25 µg/mL), followed by S.M 50% EE, S.M 100% EE, T.S 50% EE and T.S 100% EE (90.7 ± 10.7, 94.9 ± 8.42, 95.1 ± 0.025, and 105 ± 1.46 µg/mL, respectively). Figure 2E used the ClustVis: a web tool for visualizing clustering of multivariate data using principal component analysis and heatmap. The heat map chart summarizes the results of in vitro antioxidant activities. The color of the chart [white (low) to blue (high)] was related to the concentration of the compound and its corresponding IC_50_.

### Cytotoxicity and growth inhibitory potential on human liver (HepG2) and colon (Caco-2) cancer cell lines (MTT assay)

MTT assay were performed as a three separate experiments to examine the cytotoxicity and the growth inhibitory potential of *Salix mucronata* and *Tritticum spelta*. The first experiment included the determination of the most effective fraction of *Salix mucronata* and *Tritticum spelta* as we prepared different fraction of the examined plants using solvents of increasing polarity from non-polar (hexane), chloroform, butanol and ethyl acetate to more polar solvent (ethanol). Each examined extract, which prepared in different solvents, was prepared with different concentrations (500, 400, 300, 200 and 100 µg/mL) to be checked with HepG2 and Caco-2 cancer cell lines.

**Figure 3:**
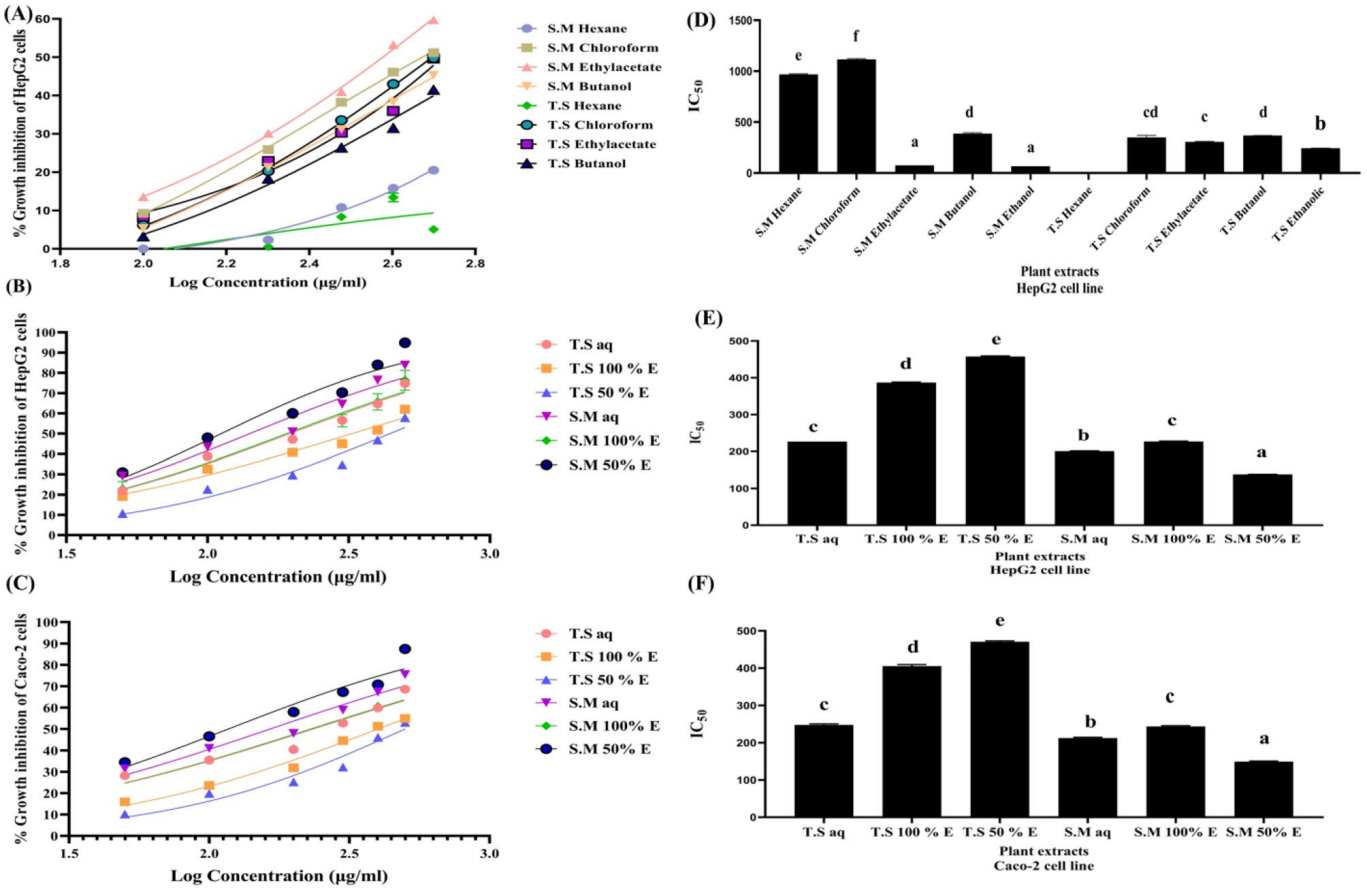
MTT results of growth inhibitory effect. Cytotoxicity effect as it was demonstrated by bar graphs; (**A**) percentage of growth inhibition of the different fractions effect of *Salix mucronata* and *Tritticum spelta* on HepG2 cancer cell line. (**B**) percentage of growth inhibition of the different aqueous, 100% EE and 50% EE of *Salix mucronata* and *Tritticum spelta* effect on HepG2 cancer cell line. (**C**) percentage of growth inhibition of the different aqueous, 100% EE and 50% EE of *Salix mucronata* and *Tritticum spelta* effect on Caco-2 cancer cell line. (**D**) Cytotoxicity of different fraction effect of *Salix mucronata* and *Tritticum spelta* on HepG2 cancer cell line. (**E**) Cytotoxicity of different aqueous, 100% EE and 50% EE of *Salix mucronata* and *Tritticum spelta* on HepG2 cancer cell line. (**F**) Cytotoxicity of different aqueous, 100% EE and 50% EE of *Salix mucronata* and *Tritticum spelta* on Caco-2 cancer cell line.

Figure 3A showed percentage of growth inhibition against log concentration of each fraction of *Salix mucronata* and *Tritticum spelta.*

Figure 3D showed the ethanolic and ethylacetate fractions of *Salix mucronata* treated HepG2 cancer cell line significantly revealed the highest anti-proliferative activity; with the lowest IC_50_ (< 76 µg/mL) (66.6 ± 0.03 and 75.3 ± 0.16 µg/mL, respectively) compared to other fractions (> 380 µg/mL) followed by *Tritticum spelta* ethanolic extract (243.12 ± 1.07 µg/mL, respectively).

The other separate second and third MTT experiment were performed as a result of previously mentioned data as ethanolic extracts has the lowest IC_50_ values. so; we focused on the study of different concentration of *Salix mucronata* and *Tritticum spelta* ethanolic extracts prepared as 100% and 50% ethanol and compare it to the prepared aqueous extract (aq E) of the same plants against two different cell lines (liver HepG2 and colon Caco-2 cancer cell lines).

The safe dose for the examined plant extracts for S.M 100% EE, S.M 50% EE, S.M aq E, T.S 100% EE, T.S 50% EE and T.S aq E were 783.02 ± 12.35, 843.53 ± 10.09, 1014.6 ± 16.9, 578.28 ± 9.3, 758.9 ± 8.9 and 834.31 ± 7.6 µg/mL, respectively.

Each examined extract was prepared with different concentrations (500, 400, 300, 200, 100 and 50 µg/mL) to be examined with HepG2 and Caco-2 cancer cell lines. As it was illustrated in Fig. 3B and C; which showed the percentage of growth inhibition against log concentration of each examined aqueous and ethanolic extract of *Salix mucronata* and *Tritticum spelta* treated liver HepG2 and colon Caco-2 cancer cell lines.

As it was illustrated in Fig. 3E and F; the lowest IC_50_ values refer to the highest anti-proloiferative activity of *Salix mucronata* and *Tritticum spelta* treated human Caco-2 and HepG2 cancer cell lines. Our study showed that S.M 50% EE significantly exhibited the highest anti-proliferative potential (especially in liver HepG2 cancer cell line) (149.14 ± 1.47 and 138.02 ± 0.5 µg/mL, respectively); and significantly followed by S.M aq E (212.48 ± 2.12 and 200.51 ± 0.98 µg/mL, respectively), T.S aq (247.6 ± 2.6 and 226.4 ± 0.07 µg/mL, respectively), S.M 100% EE (243.5 ± 2.05 and 226.7 ± 1.2 µg/mL, respectively) and T.S 100% EE (405.44 ± 4.2 and 387.19 ± 1.18 µg/mL, for human Caco-2 and HepG2 cancer cell, respectively). While T.S 50% EE significantly exhibited the lowest anti-proliferative potential compared to other examined plant extracts (470.94 ± 2.38 and 457.78 ± 1.63 µg/mL, respectively).

**Figure 4:**
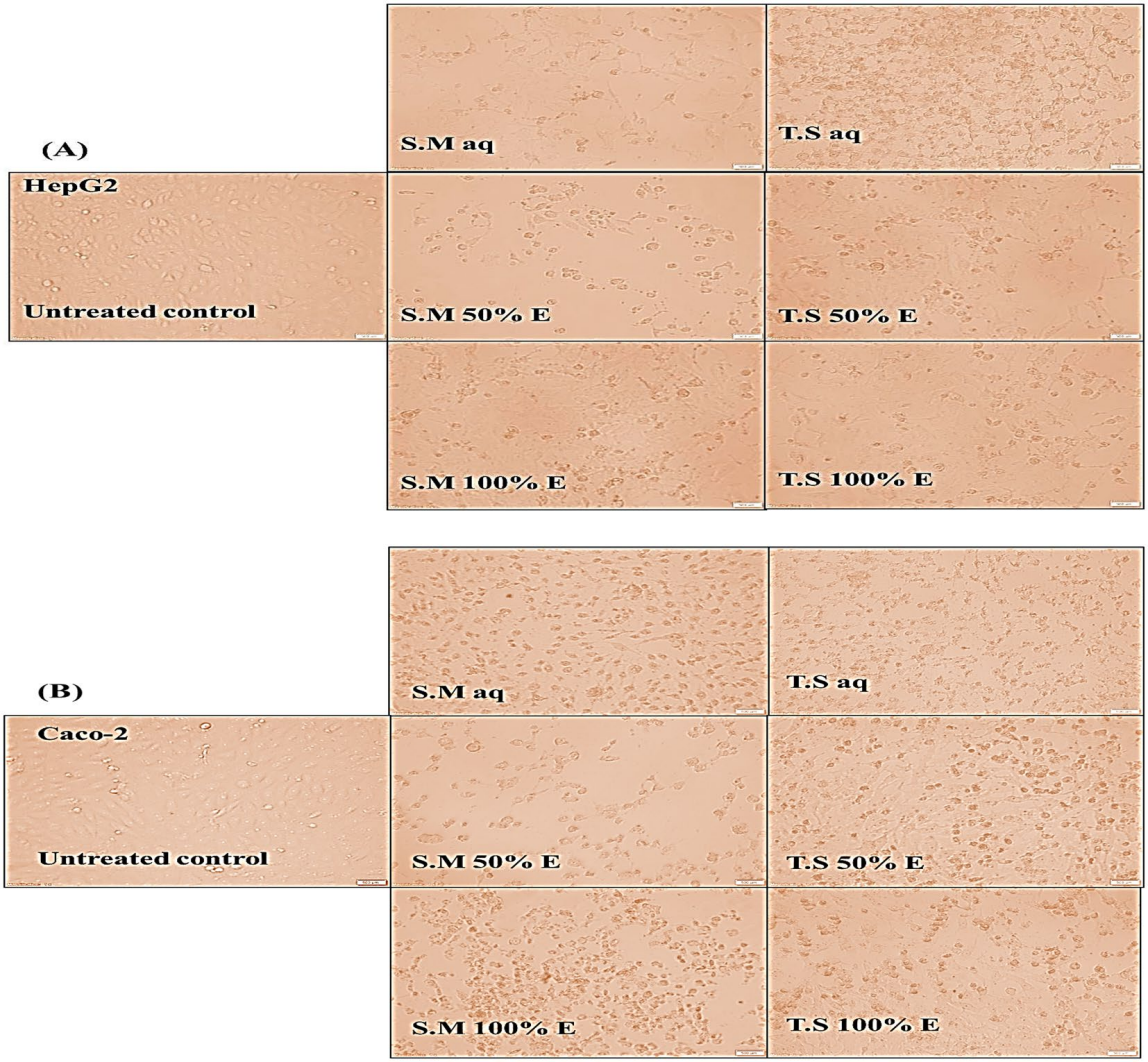
MTT results of growth inhibitory effect. Cytotoxicity effect as it was demonstrated by (**A**) Morphological alteration of different aqueous, 100% EE and 50% EE *Salix mucronata* and *Tritticum spelta* plant extracts treated HepG2 cancer cell line. (**B**) Morphological alteration of different aqueous, 100% EE and 50% EE *Salix mucronata* and *Tritticum spelta* plant extracts treated Caco-2 cancer cell line.

Figure 4A and B showed morphological alteration of different aqueous, 100% EE and 50% EE *Salix mucronata* and *Tritticum spelta* plant extracts treated HepG2 and Caco-2 cancer cell line, respectively.

### Flow cytometry analysis for the effect of aqueous and ethanolic plant extracts on human liver HepG2 and colorectal Caco-2 cancer cell lines

**Figure 5:**
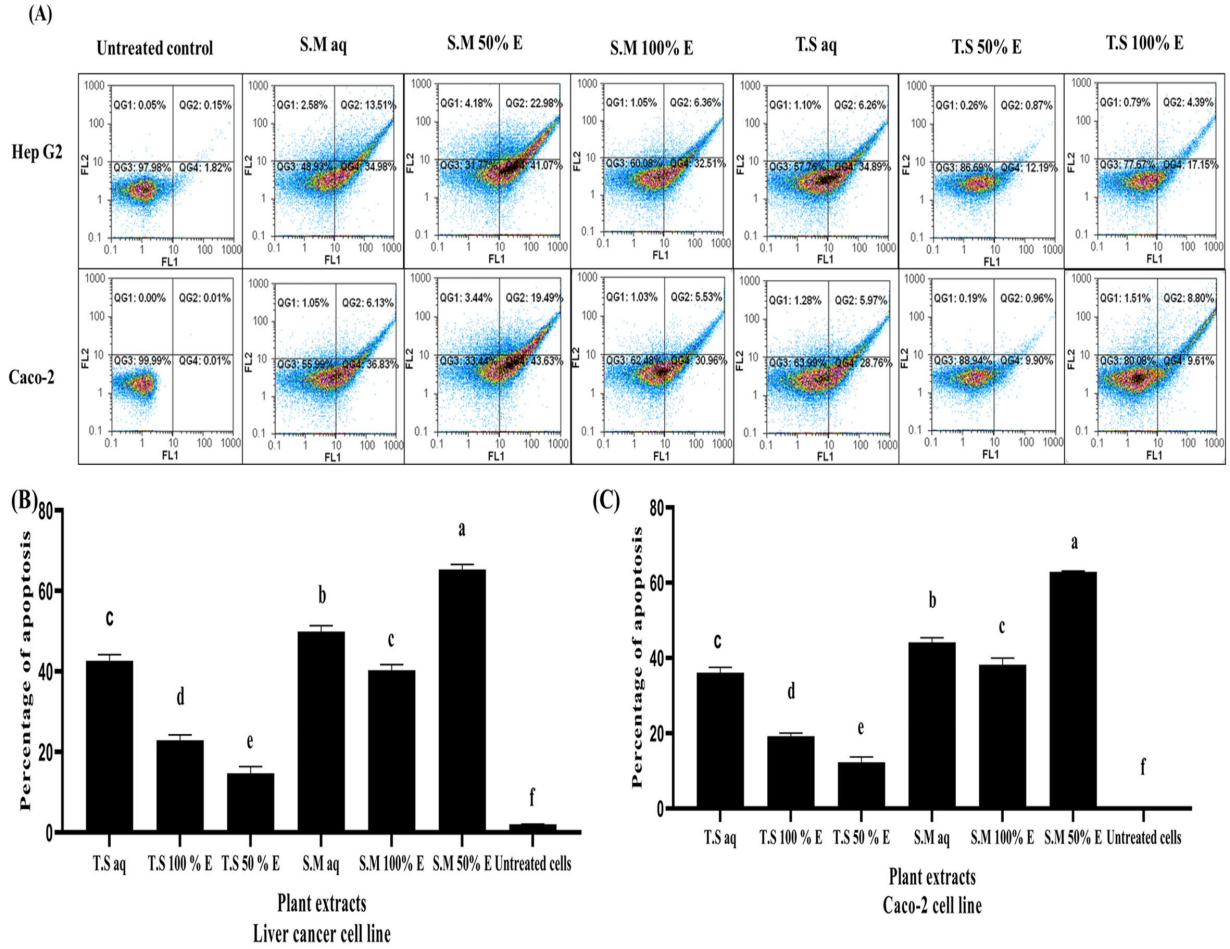
Flow cytometry analysis of the apoptotic activity of different aqueous, 100% EE and 50% EE *Salix mucronata* and *Tritticum spelta* plant extracts treated HepG2 cancer cell line and Caco-2 cancer cell line. (**A**) Dot plot charts show the apoptosis mediated anti-proliferative effect. (**B**) Represent the percentages of apoptotic population of different aqueous, 100% EE and 50% EE *Salix mucronata* and *Tritticum spelta* plant extracts treated HepG2 cancer cell line after staining with annexin-propidium iodide. (**C**) Represent the percentages of apoptotic population of different aqueous, 100% EE and 50% EE *Salix mucronata* and *Tritticum spelta* plant extracts Caco-2 cancer cell line after staining with annexin-propidium iodide. All data are expressed as mean ± SEM. Different letters are significantly different at p ≤ 0.05.

This assay is used to determine the percentage of apoptotic HepG2 and Caco-2 cancer cells after treatment with the examined extracts (aqueous, 50% and 100% EE) as compared to untreated cells. Apoptosis is detected initially by staining the cells with Annexin V which bind with phosphatidylserine. Cancer cells which are at late apoptosis stage were stained with both annexin V and propidium iodide solution. As illustrated in Fig. 5A–C; Flow cytometry analysis showed that S.M 50% EE significantly increase the number of total apoptotic cells in treated HepG2 and Caco-2 cancer cell lines (65.31 ± 1.25 and 62.87 ± 0.239, respectively) when compared to untreated cells (2.025 ± 0.05 and 0.025 ± 0.004, respectively) followed by S.M aq E (49.9 ± 1.4 and 44.17 ± 1.2, respectively), T.S aq E (42.65 ± 1.5 and 36.11 ± 1.38, respectively), S.M 100% EE (40.28 ± 1.41 and 38.23 ± 1.739) and T.S 100% EE (22.9 ± 1.36 and 19.21 ± 0.8, respectively). T.S 50% EE significantly showed the lowest apoptotic activity on HepG2 and Caco-2 cells (14.71 ± 1.65 and 12.27 ± 1.414, respectively); while the other examined plant extracts showed moderate apoptotic activity.

### The effect of the examined plant extracts on *p53, BCL2, Cyclin D, MMP9 *and* VEGF* expression levels in human liver HepG2 and colorectal Caco-2 cancer cell lines

**Figure 6:**
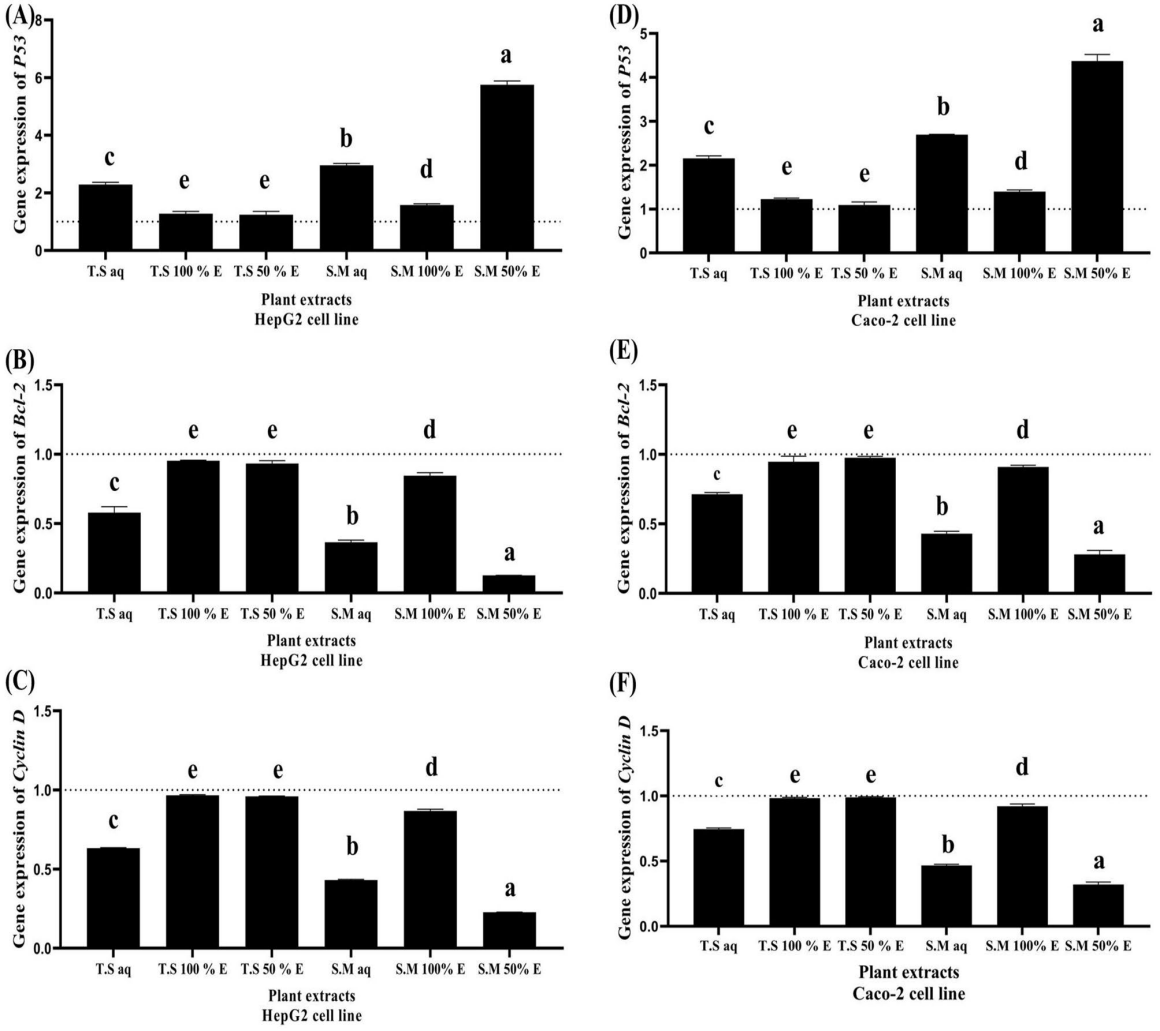
The effect of different aqueous, 100% EE and 50% EE *Salix mucronata* and *Tritticum spelta* plant extracts treated HepG2 and Caco-2 cancer cell lines on *P53*, *BCL2* and *Cyclin D* expression level (**A**) *p53* expression level in treated HepG2 cancer cell line (**B**) *BCL2* expression level in treated HepG2 cancer cell line (**C**) *Cyclin D* expression level in treated HepG2 cancer cell line (**D**) *p53* expression level in caco-2 cancer cell line (**E**) *BCL2* expression level in caco-2 cancer cell line (**F**) *Cyclin D* expression level in caco-2 cancer cell line.

As shown in Fig. 6A and D; *p53* as a significant tumor suppressor is closely associated with apoptosis and the differentiation of cancer cells. S.M 50%EE treated HepG2 and Caco-2 cell lines significantly upregulated *p53* expression level by more than five folds (5.7 ± 0.13 for HepG2 and 4.37 ± 0.14 for Caco-2, respectively). Followed by S.M aq treated HepG2 and Caco-2 cell lines which significantly upregulated *p53* expression level by three folds (2.9 ± 0.06 for HepG2 and 2.9 ± 0.062 for Caco-2). T.S aq and S.M 100% EE treated HepG2 and Caco-2 cell lines significantly upregulated *p53* expression level by two folds (2.28 ± 0.078 for HepG2 and 2.15 ± 0.05 for Caco-2 versus 1.58 ± 0.03 for HepG2 and 1.39 ± 0.04 for Caco-2, respectively). While T.S 100%EE and T.S 50%EE treated HepG2 and Caco-2 cell lines significantly failed to upregulate *p53* expression level (1.28 ± 0.07 and 1.2 ± 0.024 versus 1.24 ± 0.11 and 1.09 ± 0.06, respectively).

As shown in Fig. 6B and E; where *BCL2* is overexpressed in cancer cells, which may inhibit the pro-apoptotic signals, allowing the cancer cell to survive under stressful conditions. S.M 50% EE treated HepG2 and Caco-2 cell lines significantly downregulated *BCL2* expression level by more than ten folds in HepG2 cancer cells and by five folds in Caco-2 cancer cells (0.12 ± 0.001 for HepG2 and 0.28 ± 0.02 for Caco-2, respectively). Followed by S.M aq and T.S aq treated HepG2 and Caco-2 cell lines which significantly downregulated *BCL2* expression level by more than two folds (0.36 ± 0.014 for HepG2 and 0.42 ± 0.017 for Caco-2 versus 0.5 ± 0.042 for HepG2 and 2.9 ± 0.062 for Caco-2, respectively). T.S 50% EE, T.S 100% EE and S.M 100% EE treated HepG2 and Caco-2 cell lines significantly failed to downregulate *BCL2* expression level (0.93 ± 0.02 for HepG2 and 0.97 ± 0.01 for Caco-2 versus 0.95 ± 0.004 for HepG2 and 0.94 ± 0.04 for Caco-2 versus 0.84 ± 0.02 for HepG2 and 0.9 ± 0.01 for Caco-2, respectively).

As shown in Fig. 6C and F; the *Cyclin D1* carries out a central role in the pathogenesis of cancer. S.M 50%EE treated HepG2 and Caco-2 cell lines significantly downregulated *Cyclin D1* expression level by more than five folds in HepG2 cancer cells and by three folds in Caco-2 cancer cells (0.22 ± 0.0008 for HepG2 and 0.32 ± 0.018 for Caco-2, respectively). Followed by S.M aq and T.S aq treated HepG2 and Caco-2 cell lines which significantly downregulated *Cyclin D1* expression level by more than two folds (0.43 ± 0.004 for HepG2 and 0.46 ± 0.008 for Caco-2 versus 0.63 ± 0.004 for HepG2 and 0.74 ± 0.009 for Caco-2, respectively). T.S 50% EE, T.S 100% EE and S.M 100% EE treated HepG2 and Caco-2 cell lines significantly failed to downregulate *Cyclin D1* expression level (0.95 ± 0.002 for HepG2 and 0.98 ± 0.004 for Caco-2 versus 0.96 ± 0.004 for HepG2 and 0.98 ± 0.006 for Caco-2 versus 0.86 ± 0.01 for HepG2 and 0.92 ± 0.018 for Caco-2, respectively).

**Figure 7:**
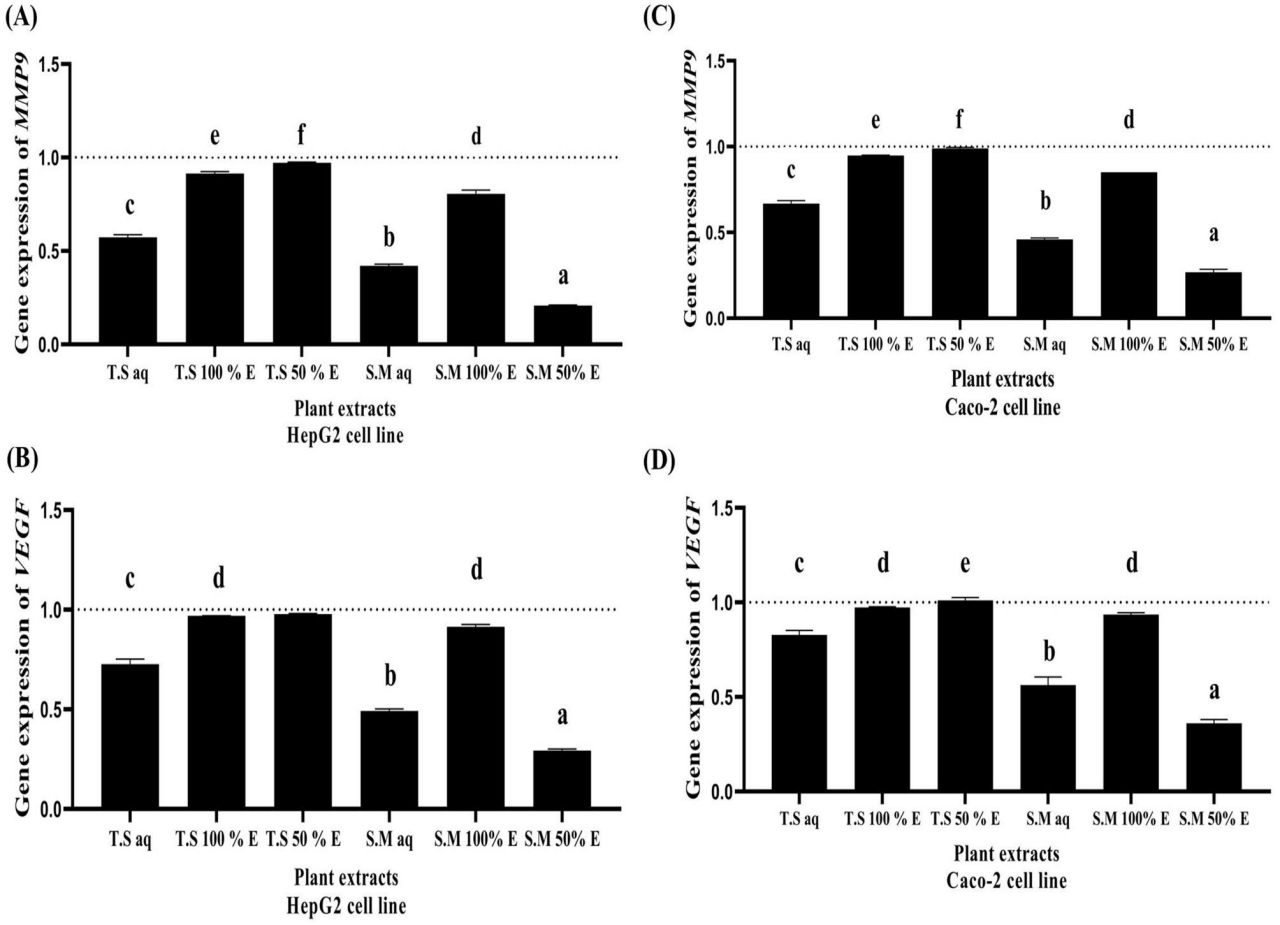
The effect of different aqueous, 100% EE and 50% EE *Salix mucronata* and *Tritticum spelta* plant extracts treated HepG2 and Caco-2 cancer cell lines on *MMP9* and *VEGF* expression level (**A**) *MMP9* expression level in treated HepG2 cancer cell line (**B**) *VEGF* expression level in treated HepG2 cancer cell line (**C**) *MMP9* expression level in treated caco-2 cancer cell line (**D**) *VEGF* expression level in caco-2 cancer cell line. All data are expressed as mean ± SEM. Different letters are significantly different at p ≤ 0.05.

As shown in Fig. 7A and C; Matrix metalloproteinases (*MMPs*) play a crucial role in tumor invasion and metastasis because of their ability to degrade extracellular matrix proteins. S.M 50%EE treated HepG2 and Caco-2 cell lines significantly downregulated *MMPs* expression level by more than five folds in HepG2 cancer cell lines and by four folds in Caco-2 cancer cell lines (0.2 ± 0.002 for HepG2 and 0.26 ± 0.017 for Caco-2, respectively). Followed by S.M aq and T.S aq treated HepG2 and Caco-2 cell lines which significantly downregulated *MMPs* expression level by more than two folds (0.42 ± 0.008 for HepG2 and 0.45 ± 0.009 for Caco-2 versus 0.57 ± 0.014 for HepG2 and 0.66 ± 0.016 for Caco-2, respectively). T.S 50% EE, T.S 100% EE and S.M 100% EE treated HepG2 and Caco-2 cell lines significantly failed to downregulate *MMPs* expression level (0.97 ± 0.003 for HepG2 and 0.98 ± 0.006 for Caco-2 versus 0.91 ± 0.009 for HepG2 and 0.94 ± 0.002 for Caco-2 versus 0.8 ± 0.02 for HepG2 and 0.85 ± 0 for Caco-2, respectively).

As shown in Fig. 7B and D; *VEGF* is one of the most important angiogenic factors and is an endothelial cell-specific mitogen. S.M 50%EE treated HepG2 and Caco-2 cell lines significantly downregulated *VEGF* expression level by more than four folds in HepG2 cancer cell lines and by three folds in Caco-2 cancer cell lines (0.29 ± 0.008 for HepG2 and 0.36 ± 0.019 for Caco-2, respectively). Followed by S.M aq and T.S aq treated HepG2 and Caco-2 cell lines which significantly downregulated *VEGF* expression level by more than two folds (0.49 ± 0.01 for HepG2 and 0.56 ± 0.04 for Caco-2 versus 0.72 ± 0.02 for HepG2 and 0.82 ± 0.02 for Caco-2, respectively). T.S 50% EE, T.S 100% EE and S.M 100% EE treated HepG2 and Caco-2 cell lines significantly failed to downregulate *VEGF* expression level (0.97 ± 0.002 for HepG2 and 1.01 ± 0.01 for Caco-2 versus 0.96 ± 0.0007 for HepG2 and 0.97 ± 0.003 for Caco-2 versus 0.91 ± 0.011 for HepG2 and 0.93 ± 0.01 for Caco-2, respectively).

### High performance liquid chromatography (HPLC) assay to study the bioactive compounds of the most potent plant extract (*Salix mucronata*)

**Figure 8:**
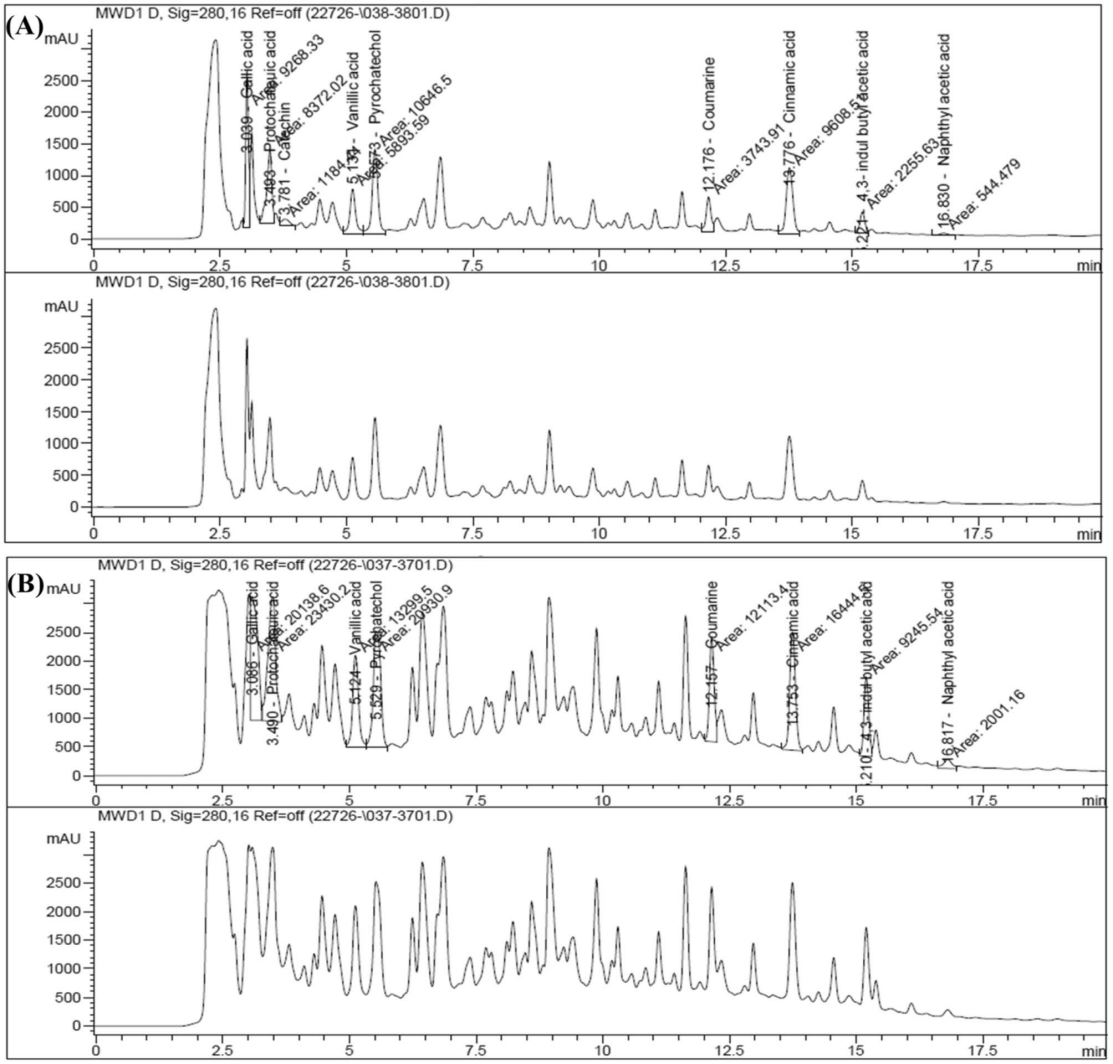
HPLC analysis of polyphenolic compounds (**A**) HPLC analysis of bioactive compounds in the *Salix mucronata* 50% EE and (**B**) HPLC analysis of bioactive compounds in the *Salix mucronata* 100% EE.

According to the previously mentioned results; S.M 50% EE and S.M 100% EE showed the highest anti-proliferative, anti-oxidant and apoptotic activities against cancer cells. HPLC analysis was performed for identification and quantification of bioactive compounds in S.M 50% and 100% EE. The major bioactive compounds that were found in S.M 50% EE and S.M 100% EE were coumarine, cinnamic acid, -4,3-indul butyl acetic acid and naphthyl acetic acid. They also contain other minor bioactive compounds such as protochateuic acid, gallic acid, vanillic acid and pyrochatechol. Catechin is detected in S.M 50% EE while it wasn’t detected in S.M 100% EE (Fig. 8, and Table 2 in supplementary files).

## Discussion

Natural chemicals originating from plants are of great interest in the ongoing search for safer and more efficient treatments than the use of drugs. Secondary metabolites known as plant phenolic have attracted attention as possible anti-cancer agents. Phenolic compounds have a significant potential as cytotoxic anti-cancer drugs since they target several components of cancer, increase apoptosis, and decrease proliferation (angiogenesis, growth, differentiation, and metastasis). In numerous in vitro and in vivo investigations, phenolic acids (benzoic and cinnamic acids) have been linked to powerful anticancer properties. Additionally, the therapeutic effects of phenolic acids are strengthened by their function as epigenetic regulators and supporters of unfavorable side effects or resistance linked to traditional anticancer therapy^14,15^.

It is widely believed that excessive cellular oxidative stress plays a significant role in the development of pathophysiological diseases and cancer. To prevent damage to DNA, proteins, and lipids, healthy cells employ a variety of processes to control intracellular levels of reactive oxygen species (ROS) and overall redox equilibrium. This in turn shows the potential application of redox regulation as a therapeutic approach for the treatment of cancer^14^. Regulatory proteins including kinases (*MAPK* and *PI3K/Akt*), transcription factors (*Nrf2*, *AP-1*, *NF-B*, *STAT3*, and *P53*), molecular chaperones, and cytoskeletal proteins can all have their physiological function altered by ROS^8^.

The six main hallmarks of cancer progression include uncontrolled cell proliferation and differentiation, replicative immortality, angiogenesis promotion, enhanced proliferative signaling, resistance to cell death, and metastatic invasion. To regulate and cure cancer, it is therefore necessary to focus on the molecular pathways while aiming to restore the altered equilibrium. Our novel in vitro study is to investigate two edible plant extracts (*Salix mucronata* and *Triticum spelta*) according to their phytochemical composition, anti-oxidant, anti-proliferative, radical scavenging, metal chelating activities and their possible affected molecular pathways.

Authors examined two edible ethanolic plant extracts (50% and 100% EE) effects on human liver HepG2 and colorectal Caco-2 cancer cell lines. MTT assay was performed for investigating the anti-proliferative effect of *Salix mucronata* and *Tritticum spelta* plant extracts on HepG2 and Caco-2 cancer cell lines. The study revealed that the ethanol and ethylacetate fractions of *Salix mucronata* and *Tritticum spelta* extract showed the highest anti-proliferative activity (IC_50_ < 76 µg/mL) compared to its other fractions (IC_50_ > 380 µg/mL). Consequently, authors focused on the study of different concentration of *Salix mucronata* and *Tritticum spelta* ethanolic extract (as it was the most potent fraction, prepared as 100% and 50%), and compare it with the prepared aqueous extract of the same plants. It has been shown that ethanolic extracts of *Salix mucronata* and *Tritticum spelta* contain high phenolic and flavonoid contents. Also, it has been known that phenolic compounds have proved their ability to act as anti-oxidants by donating a hydrogen atom. Furthermore, flavonoids are phenolic substances found in plants that prevent lipid oxidation by scavenging radicals or through other mechanisms such as singlet oxygen quenching, metal chelation, and lipoxygenase inhibition^10^.

The antioxidant-dependent anti-proliferative properties, especially in *Salix mucronata*, are significantly caused by the presence of high levels of total phenolic content in examined S.M 100% EE followed by S.M 50% EE; that would be agreed with El-Sayed et al.^16^. In addition; *Salix mucronata* and *Tritticum spelta* prepared as 50% and 100% EE have the same flavonoids content.

The redox characteristics of phenolics, which enable them to function as reducing agents, hydrogen donors, and singlet oxygen quenchers, are primarily responsible for the antioxidant actions of phenolics^17^. It has been demonstrated that the genesis, advancement, and spread of malignancies in cells in vitro and in animals in vivo can be inhibited or attenuated by both monophenolic and polyphenolic chemicals found in a wide variety of plant foods, spices, and drinks. Cell signaling cascades, including kinases and transcription factors, which control the expression of genes involved in cell cycle arrest, cell survival, and apoptosis, or programmed cell death, are among the many biological pathways that phenolics modify to elicit these anticancer effects. The inhibitory effects of phenolics on the stress-activated *NF-KB* and *AP-1* signal cascades in cancer cells, which are thought to be important therapeutic targets, have received a lot of attention^18^.

Phenolics can improve the body's immune system's capacity to identify and eradicate cancer cells as well as limit angiogenesis, the formation of new blood vessels required for tumor development. Additionally, they lessen the adhesion and invasiveness of cancer cells, which lowers their potential for metastasis. Another significant effect of plant phenolics that calls for further study is the enhancement of the effectiveness of conventional chemo- and radio-therapeutic treatment regimens and the prevention of resistance to these treatments. Plant phenolics should be further studied because they seem to have both cancer prevention and therapy potential^18^. Among all ROS, hydroxyl radical has been known to be the most powerful free radical. It may cause lipid oxidation and enormous biological damage leading to cancer^14^. S.M 100% EE, S.M 50% EE, T.S 100% EE and T.S 50% EE have non-significant total anti-oxidant effect, ferric reducing power, hydroxyl radical and superoxide anion scavenging activities. The ferric reducing power method does not involve free radicals, but involves reduction of the ferricyanide (Fe^+3^) to ferrocyanide (Fe^+2^), by the reducing agent^15^. The presence of high phytochemical content results in significant potent reducing power and chelating activity in the examined ethanolic extracts and they were more potent than vitamin C. They exert their action by breaking the free radical chain through donation of a hydrogen atom. Examined plant extracts showed reducing and free-radical scavenging properties, but they also have metal chelating properties that can stop ROS from being produced by Cu^+2^ and Fe^+2^ catalysts (Fenton reaction). According to the iron chelating activity; S.M 50% EE showed a significant highest activity followed by the other examined plants extracts, in contrast to copper chelating activity; S.M 100% EE showed a significant highest activity followed by T.S 100% EE, S.M 50% EE and T.S 50% EE, respectively.

*Salix mucronata* and *Tritticum spelta* ethanolic extracts contain a significantly high flavonoid content, accordingly, S.M 50% EE, T.S 100% EE and T.S 50% EE showed the highest DPPH scavenging activity, followed by S.M 100% EE, and they are more potent compared to vitamin C. This indicated the potential hydrogen donating ability to reduce DPPH. Regarding the nitric oxide scavenging activities of each examined extract, vitamin C, T.S 50% EE and T.S 100% EE showed a significantly high scavenging activities followed by S.M 50% EE and S.M 100% EE.

Based on MTT results, S.M 50% EE with the lowest IC_50_ is capable for inhibiting growth of the investigated human colorectal (Caco-2) and liver (HepG2) cancer cell lines, compared to other prepared plant extracts. The anti-proliferative effect of each examined plant extract was arranged from the lowest IC_50_ to the highest as follow; S.M 50% EE followed by S.M aq, S.M 100% EE, T.S aq, T.S 100% EE and T.S 50% EE, respectively. Significantly; this powerful anti-proliferative effect is also supported with severe morphological alterations (cell shrinking and loss of normal spindle shape) of the treated human liver (HepG2) and colorectal (Caco-2) cancer cells in comparison with the untreated cells. *Salix mucronata* have a long history of usage in folk medicine as an anti-leukemia, analgesic, and anti-pyretic herbal remedy. Alcoholic -glucoside salicin, which can be dissolved in both water and ethanol, is the major active component in Salix leaves. Salicin may be the main ingredient causing the anti-tumor activity, although additional metabolites may make the willow extract more effective than salicin alone^19^.

Flow cytometry analysis was used to identify the apoptotic features after labeling the cells with Annexin V and propidium iodide (PI) solution. It is based on the idea that normal cells express phosphatidyl serine in the inner membrane, which is the side that faces the cytoplasm, making it hydrophobic by nature. However, during apoptosis, the inner membrane flips over to become the outer membrane, exposing the phosphatidyl serine. Propidium iodide stains necrotic cells, which have leaky DNA content that helps to distinguish the apoptotic and necrotic cells, while Annexin V detects the exposed phosphatidyl serine of early apoptotic cells. Apoptotic cells are stained with PI and nuclear annexin V at a late stage^20^.

This assay is used to determine the percentage of apoptotic HepG2 and Caco-2 cancer cells after treatment with the investigated plant extracts (aqueous, 50% and 100% EE) compared to untreated cells. It has been shown that S.M 50% EE treated HepG2 and Caco-2 cancer cells significantly increases the number of total apoptotic cells with increase of *p53* gene expression and downregulation of *BCL2*, *Cyclin D*, *MMP9* and *VEGF* signaling pathway by more than fivefold followed by S.M aq, T.S aq and S.M 100% EE with a moderate apoptotic activity (from two folds up to five fold). In contrast, T.S 100% EE and T.S 50% EE treated HepG2 and Caco-2 cancer cells significantly have the lowest percentage of apoptotic cells and failed in upregulating *p53* gene expression and downregulation of *BCL2*, *Cyclin D*, *MMP9* and *VEGF* signaling pathway. *p53* inactivation is linked to about 50% of cancer cases. Apoptosis, senescence, angiogenesis, cell cycle regulation, cellular differentiation, antioxidant responses and DNA metabolism are some of the major biological roles of *p53*. In contrast, *p53* offers irreversible apoptotic programs by activating the *Bax* gene, a crucial member of the *BCL2* family, then *Bax* attaches to *BCL2*, causing the synthesis of apoptotic mediators (like caspase) to be activated. Therefore, *BCL2* targeting via *p53* offers effective cancer treatment options^6^. When *BCL2* is overexpressed in cancer cells, it suppresses pro-apoptotic signals, allowing the cancer cell to survive in stressful situations^21^. *Cyclin D1* functions as a cell cycle regulator; and its overexpression promotes tumor growth by allowing unregulated cellular proliferation^22^.

Angiogenesis is a crucial step in the process of systemic metastasis and is significant in the formation and progression of solid tumors. One of the most important angiogenic factors is *VEGF*^23^. In many tumor cells, higher expression of matrix metalloproteinases (*MMPs*), particularly *MMP-2* and *MMP-9*, is linked to enhanced metastatic potential^24^. Regarding their ability to destroy extracellular matrix proteins, matrix metalloproteinases (*MMPs*) play a critical role in tumor invasion and the creation of metastatic forms^25^.

Moreover, HPLC analysis was carried out for S.M 50% EE and S.M 100% EE to compare their different composition. Obtained data revealed that *Salix mucronata* demonstrated effective biological anti-proliferative and anti-oxidant activity in all its prepared form especially 50% and 100% EE. HPLC analysis of polyphenolic content of S.M 50% EE and S.M 100% EE revealed that the content of the major bioactive compounds were naphthyl acetic acid, 4,3-indul butyl acetic acid, cinnamic acid, and coumarine. They also contain minor bioactive compounds such as protochateuic acid, gallic acid, vanillic acid and pyrochatechol. Catechin is detected in S.M 50% EE while it wasn’t detected in S.M 100% EE. Therefor; S.M 50% EE showed a slight increase in polyphenolic content than S.M 100% EE; that may be the reason for being more effective. The presence of catechin in S.M 50% EE rather than S.M 100% EE, gives advantage to S.M 50% EE to be more effective than S.M 100% EE as Catechins plays a role in either cancer therapy or cancer prevention in vivo and in vitro via the inhibition of *c-Jun* and *ERK1/2* phosphorylation and the suppression of *VEGF*-dependent angiogenesis^26^.

The antioxidant, antibacterial, anticancer, antiulcer, antidiabetic, antifibrotic, antiviral, anti-inflammatory, analgesic, cardiac, antiaging, hepatoprotective, neurological, and nephroprotective efficacy are just a few of the biological and pharmacological activities of protocatechuic acid, gallic acid, vanillic acid and hydroxycinnamic acids. These phenolic acids act by activating *JNK* and *p38-MAPK* pathway, stimulating the cell death in cancer; also they show anti-proliferative and antiapoptotic effect via decreasing *BCL2* expression, activating the *Fas/FasL* pathway, upregulating *caspase-3*, *Bax,* suppressing *MMP-2* and *VEGF* dependent *Akt/MMP2*, and subsequently reducing cancer cells ability to survive, proliferate, and invade^27,28^.

## Conclusion

Phytochemicals are currently gaining great importance in both cancer prevention and treatment due to their antioxidant, anti-proliferative, anti-angiogenic, pro-apoptotic, and anti-cancer properties. Therefore, our study was directed to investigate the anti-proliferative effect of two edible plant extracts (*Salix mucronata* and *Tritticum spelta*). In vitro evaluation of phyto-chemical content, anti-oxidant, anti-proliferative, radical scavenging and anti-cancer activities showed that *Salix mucronata* treated was found to be more effective than *Triticum spelta. Salix mucronata* illustrated their anti-proliferative effect against human colorectal (Caco-2) and liver (HepG2) cancer cells via blocking the production of reactive oxygen species, having a metal chelating activity, enhancing *p53* expression level, and downregulating *Cyclin D, MMP9, VEGF* and *BCL2* gene expression. *Salix mucronata* represents a promising antioxidant-dependent pro-apoptotic agent for eradicating liver and colon cancer cells as it has salicin as an active compound which is responsible for its anti-proliferative activity.

## Methods

### Chemicals

Folin–Ciocalteau reagent, gallic acid (GA), rutin (RU), sulfanilamide, naphthylethylenediamine dihydrochloride, Diphenyl–α-picrylhydrazyl (DPPH), nitroblue tetrazolium (NBT), riboflavin, and vitamin C were supplied from Riedel-de Haën, Germany. DMEM medium and fetal bovine serum (FBS) were obtained from Lonza (USA). Other chemicals were obtained with a high grade from Sigma-Aldrich (St. Louis, MO, USA). Primers for P53, *BCL2*, Cyclin D, MMP9 and VEGF were purchased from Bioneer, Korea as shown in Table 1 in supplementary materials.

### Preparation of *Salix mucronata* and *Tritticum spelta* plant extract using a separation funnel method

Ethical approval for this study was obtained from the local ethics committee of the faculty of science, university of Alexandria, Egypt. Also, all experiments were performed in accordance with relevant guidelines and regulations. Moreover, there are no rare plants used in this study. Two plants were separately collected from local market and identified at Botany and Microbiology Department, Faculty of Science, Alexandria University. The plants were *Salix mucronata* and *Tritticum spelta*.

To study the most effective method of extraction, five different solvents (n-hexane, chloroform, n-butanol, ethyl acetate and ethanol) were used. Fractionation begins by moistening or complete dissolution of plants crude extract with 250 mL of water. This is followed by transfer into a separating funnel, shaken, and allowed to settle. Furthermore, 250 mL of n-hexane, the least polar solvent was added and shaken. The content lets to settle, and the bottom of the separating funnel opened to remove the aqueous layer. The remaining content in the separating funnel was poured into a clean container to get n-hexane fraction. Equal volume of n-hexane was added again, shaken, and separated. This process continued until after adding n-hexane and shaken no reasonable quantity of extract appeared to move into the n-hexane portion. Similar cycle was performed for chloroform, acetone, n-butanol, ethyl acetate and ethanol to get chloroform, acetone, n-butanol, ethyl acetate and ethanol fractions. The remaining portion left after the fractionation is termed as residual aqueous fraction (RAF) as the crude extract was first dissolved in water^29^.

### Ethanolic extract preparation

10 mg of *Salix mucronata* dried leaves or *Tritticum spelta* were minced with (100 mL) 50% or 100% ethanol separately for one day, and then filtered off. Each filtrate was collected, evaporated under reduced pressure using rotary evaporator (Telstar) separately, then lyophilized and weighed. The obtained ethanolic extract (powder), that containing active ingredient, was dissolved at concentration of 1 mg/mL distilled water.

### Phytochemical analysis

Briefly, the total phenolic contents of each ethanolic extract or serial concentrations of GA (standard) were mixed with Folin–Ciocalteau reagent and sodium carbonate. Phenolic concentration (mg/100 g plant tissue) was estimated using the standard curve of GA^30^.

The total flavonoid contents of each ethanolic extract or serial concentrations of RU (standard) were mixed with sodium nitrite and aluminum chloride solution. Flavonoid concentration (mg/100 g plant tissue) was estimated using the standard curve of RU^31^.

### In vitro evaluation of antioxidant activity

The total antioxidant capacity of each examined ethanolic plant extracts or different concentrations of vitamin C (6.25, 12.5, 25, 50, 100 and 200 µg/mL), as standard, were performed according to Tyagi study. Results were expressed as ascorbic acid equivalents in mg/g using the standard curve of vitamin C^32^.

Ferric reducing power was examined for ethanolic plant extract or positive control ascorbic acid by adding phosphate buffer and potassium ferricyanide to them according to Oyaizu method^15^. The results were expressed as EC_50_ (µg/mL) and were calculated using Graphpad Instat software.

Metal chelating activities included the determination of iron and copper chelating activity of each examined ethanolic plant extract were determined by Minotti^33^ and Saiga^34^ methods, respectively. The effective concentration (EC_50_) value (at 50% iron or copper chelation) of each examined plant extract was calculated using Graphpad Instat software.

### Free radicals scavenging activity assays

DPPH scavenging activity assay of each ethanolic plant extract was estimated using methanolic solution of DPPH reagent^35^. Nitric oxide scavenging activity was estimated using Sodium nitroprusside and Griess reagent^36^. Hydroxyl radical scavenging activity of each examined ethanolic plant extract was measured by salicylic acid method using vitamin C as standard^37^.

Superoxide anion scavenging activity was performed using riboflavin, nitro blue tetrazolium and vitamin C as standard^38^.

### In vitro evaluation of anti-proliferative activity

#### Determination of growth inhibitory potential using the MTT (3-[4,5-dimethylthiazol-2-yl]-2,5 diphenyl tetrazolium bromide) assay on different cancer cell lines

MTT assay was performed three times separately, the first MTT experiment was performed to evaluate the anti-proliferative effect of each examined plant extract obtained from 5 different solvents (n-hexane, chloroform, n-butanol, ethyl acetate and ethanol). That was assayed using human liver cancer cell line (Hep G2), to find the most effective fraction according to Mosmann^39^. Each plant extract were prepared with the following concentration 500, 400, 300, 200 and 100 µg/mL.

On the contrary, the second and third separate MTT experiments were performed to evaluate the anti-proliferative effect of aqueous, 50% and 100% ethanolic extract of *Salix mucronata* and *Triticum spelta* that were assayed using human liver (HepG2) and colorectal cancer cells (Caco-2), respectively. Each plant extract was prepared with the following concentrations; 500, 400, 300, 200, 100 and 50 µg/mL. Each cancer cell line has been cultured in DMEM (Lonza, USA) supplemented with 10% FBS (GEBCO, USA). Cells (4 × 10^3^ cells/well) were seeded in sterile 96-well plates. After 24 h, serial concentrations of *Tritticum spelta* and *Salix mucronata* different fractions were incubated with each cancer cell lines separately for 72 h at 37 °C in 5% CO_2_ incubator. Cell viability was assayed by MTT method^39^. The half maximal inhibitory concentration (IC_50_) values were calculated using the Graphpad software. Furthermore, cellular morphological changes before and after treatment with the examined compounds were investigated using phase contrast inverted microscope with a digital camera.

### Evaluation of pro-apoptotic activity in plant extract-treated human liver (HepG2) and colorectal cancer cells (Caco-2)

#### Flow cytometry analysis

Aqueous, 50% and 100% ethanol *Tritticum spelta* and *Salix mucronata* plant extracts were tested for their pro-apoptotic activity. The annexin V apoptosis detection kit for flow cytometry (Sigma-Aldrich, MO, USA) was used. The annexin V assay was carried out in conjunction with propidium iodide (PI) staining. HepG2 and Caco-2 cancer cell lines were cultured for 24 h in a 25 cm^2^ culture flask (1 × 10^6^ cells/well) with 66.6 μg/mL (the lowest IC_50_) of the investigated extracts. After 72 h, cells were harvested by trypsinization and centrifuged at 1000 rpm for 5 min and then re-suspended in 1 × binding buffer prior to staining with 5 μL of annexin V and 10 μL of propidium iodide solution for 15 min at room temperature. The apoptosis-dependent anti-proliferative effect was determined by quantification of annexin-stained apoptotic cells using the FITC signal detector (FL1) against the phycoerythrin emission signal detector (FL2)^20^.

### The effect of the different plant extracts on *P53, BCL2, Cyclin D, MMP9 *and *VEGF* expression levels

Quantitative real time polymerase chain reaction (qRTPCR) was used to study the effect of *Tritticum spelta* and *Salix mucronata* plant extract on *p53*, *BCL2*, *Cyclin D*, *MMP9* and *VEGF* gene expression. Total RNAs were extracted from the untreated and the treated HepG2 and Caco-2 cancer cell lines using Gene JET RNA Purification Kit (Thermo Scientific, USA). Then cDNA was synthesized utilizing cDNA Synthesis Kit (Thermo Scientific, USA). Real time PCR was performed using SYBR green master mix and specific primers which are shown in Table 1 in supplementary materials. The 2^−ΔΔCT^ equation was used to calculate the change in gene expressions in the treated cancer cells relative to untreated cancer cells. The expression of target genes was calculated using the comparative Ct method (threshold cycle number at cross-point between amplification plot and threshold). The CT values of each target gene were normalized to that of β-actin according to manufacturer’s instructions and the change in expression (2^−ΔΔCT^) was calculated.

### HPLC analysis for identification and quantification of phenolic content in the most active extracts

HPLC analysis was performed in the City of Scientific Research and Technological Applications, Central Lab. New Borg El-Arab City, Alexandria, Egypt. Twenty microliters of the most effective plant extract (*Salix mucronata* 50% and 100% EE) were separated on 150 mm × 4.6 mm, 5 μm Eclipse XDB–C18 column (Agilent Technologies, Palo Alto, CA, USA). The separation was at a flow rate of 0.75 mL/min and at 320 nm. The mobile phase was 1% formic acid: acetonitrile: 2-propanol (70:22:8), pH 2.5^40^.

### Data analysis

The data are expressed as mean ± SEM and the significant values were considered at *p* < 0.05. One-way analysis of variance (ANOVA) by Duncan’s test was used for evaluating the difference between the mean values of the results obtained using plant extracts. The analysis was done for three measurements using SPSS software version 16. Heat map plots were generated by ClustVis web tool (https://biit.cs.ut.ee/clustvis/)^41^.

### Supplementary Information

Supplementary Table 1.

Supplementary Table 2.

## Data Availability

All data and materials are fully presented in the manuscript.
