# Author Correction: Potential anti-proliferative activity of *Salix mucronata* and *Triticum spelta* plant extracts on liver and colorectal cancer cell lines

**DOI:** 10.1038/s41598-023-41534-2

**Published:** 2023-09-04

**Authors:** Ghada M. Ahmad, Marwa M. Abu Serie, Mohamed S. Abdel-Latif, Tayseer Ghoneem, Doaa A. Ghareeb, Galila A. Yacout

**Affiliations:** 1 Department of Medical Laboratory Technology, Faculty of Applied Health Sciences Technology, Pharos University in Alexandria, Alexandria, Egypt; 2 Medical Biotechnology Department, Genetic Engineering and Biotechnology Research Institute (GE-BRI), City of Scientific Research and Technological Applications (SRTA-City), New Borg El-Arab, 21934 Alexandria Egypt; 3 Department of Biochemistry, Faculty of Science, Alexandria University, Alexandria, Egypt

Correction to: *Scientific Reports* 10.1038/s41598-023-30845-z, published online 07 March 2023

The original version of this Article contained an error in Affiliation 2, which was incorrectly given as ‘Department of Medical Biotechnology, Genetic Engineering and Biotechnology Research Institute, New Borg El‑Arab City, Alexandria, Egypt’. The correct affiliation is listed below:

Medical Biotechnology Department, Genetic Engineering and Biotechnology Research Institute (GE-BRI), City of Scientific Research and Technological Applications (SRTA-City), New Borg El-Arab 21934, Alexandria, Egypt.

The original Article has been corrected.

