# Supplementary material for: Potential anti-proliferative activity of Salix mucronata and Triticum spelta plant extracts on liver and colorectal cancer cell lines
Source: Sci Rep. 2023 Mar 7;13:3815. doi: 10.1038/s41598-023-30845-z (PMC9992471; doi:10.1038/s41598-023-30845-z)
Supplement: Supplementary file 1 — Supplementary Table 1. [file 41598_2023_30845_MOESM1_ESM.docx]

**Table (1): Real time PCR forward and reverse primer sequences (Supplementary materials)**

| **Gene** | **Forward primer** | **Reverse primer** |
| --- | --- | --- |
| ***p53*** | 5′-ATGTTTTGCCAACTGGCCAAG-3′ | 5′-TGAGCAGCGCTCATGGTG-3′ |
| ***BCL2*** | 5′-TCCGATCAGGAAGGCTAGAGTT-3′ | 5′-TCGGTCTCCTAAAAGCAGGC-3′. |
| ***Cyclin D*** | 5′-TACTCTGGCGCAGAAATTAGGTC3′ | 5′CTGTCTCGGAGCTCGTCTATTTG-3′ |
| ***MMP9*** | 5′-TCTATGGTCCTCGCCCTGAA-3′ | 5′-CATCGTCCACCGGACTCAAA-3′ |
| ***VEGF*** | 5′-GAGGGCAGAATCATCACGAAG-3′ | 5′CACACAGGATGGCTTGAAGA-3′ |
| ***β-actin*** | 5’-GGGAAATCGTGCGTGACATC-3′ | 5’-GCGGCAGTGGCCATCTC-3′ |
