# Supplementary material for: Potential anti-proliferative activity of Salix mucronata and Triticum spelta plant extracts on liver and colorectal cancer cell lines
Source: Sci Rep. 2023 Mar 7;13:3815. doi: 10.1038/s41598-023-30845-z (PMC9992471; doi:10.1038/s41598-023-30845-z)
Supplement: Supplementary file 2 — Supplementary Table 2. [file 41598_2023_30845_MOESM2_ESM.docx]

Table (2): HPLC analysis of bioactive compounds in the *Salix mucronata* plant extracts (50% and 100% ethanolic extract)

**(Supplementary materials)**

| **Phenolic compound** | ***Salix mucronata***  **(50% ethanolic extract)** | ***Salix mucronata***  **(100% ethanolic extract)** |
| --- | --- | --- |
|  | **µg/mL** | |
| **Gallic acid** | 3.039 | 3.086 |
| **Catechin** | 3.78 | Not detected |
| **Vanillic acid** | 5.13 | 5.124 |
| **Pyrochatechol** | 5.573 | 5.5 |
| **Coumaric acid** | **12.17** | **12.15** |
| **4,3- indul butyl acetic acid** | **15.221** | **15.21** |
| **Naphthyl acetic acid** | **16.83** | **16.817** |
| **Cinnamic acid** | **13.77** | **13.75** |
| **Protochateuic acid** | 3.49 | 3.49 |
